# The History and Capabilities of Herbal Simples

**Published:** 1890-06-14

**Authors:** W.T. Fernie


### The History and Capabilities of Herbal Simples.

By W. T. Fernie, M.D.

*(Continued from page* xxxiv.*)*

### XI.—WHITE BRYONY.

In pugilistic parlance, when Jem the Slogger gets home with his right mawley on Black Nat's sinister optic, a mouse, *i.e.*, a black eye, is the immediate consequence; and for this the remedy most approved in the Ring is a piece of raw beef quickly applied. But it would be far more useful to promptly bathe the contused part with a decoction of the white bryony root, which serves to prevent discoloration and speedily subdues the swelling. This plant abounds in English hedges, and is often called the wild vine: also it bears the name of tetterberry, from its curing a disease of the skin known as tetters. It climbs about with long straggling stalks which attach themselves by spiral tendrils, and which bear large rough palmated leaves. The insignificant pale green flowers spring in small clusters from the bottom of these leaves. The round berries are at first green, and afterwards bright red. Its name, bryony, is two thousand years old, and comes from a Greek word, bruein—to shoot forth rapidly. Botanically this plant belongs to the order of cucumbers. Chemically it contains a medicinal substance—bryonine—which is intensely bitter; also malate, and phosphate of lime, with gum, starch, and sugar. A tincture is made from the fresh root collected before the plant flowers, and this is found to be of superlative use for the relief of rheumatism, and for subduing active congestions of serous membranes. In the treatment of pleurisy it is invaluable. Four drops of the tincture may be given in a tablespoonful of cold water every two, three, or four hours.

In France the white bryony is deemed so potent and perilous, that its root is termed the devil's turnip—*navet du diable*. Our English plant, the bryonia dioica, purges as actively as colocynth when too largely administered. A certain herbal drink which is at the present time in popular esteem, and known as Hop Bitters, is reputed to owe much of its useful virtue to the rare and famous mandrake which it contains. But most probably here, as in other like instances, the root of white bryony has been substituted. The true mandrake was held in superstitious awe by the Greeks and Romans. Its root was forked, and bore some resemblance to the legs of a man, for which reason the crafty money-seekers of the past increased the similitude, and then attributed to the plant supernatural powers. Josephus describes how the mandrake was pulled up by the roots in Jewish villages, a process which would cause death to any animal or creature hearing the screams it was supposed to make. The only safe way, therefore, was to stop the ears, and then, after having tied the tail of a dog securely to the plant, to run away. When at a safe distance, by calling the dog, the coveted root would be pulled up, but the animal would fall dead on the spot. From earliest times in the East a notion has prevailed that mandrakes would remove sterility. Having this purpose in view, Rachel said to Leah (Genesis xxx. 14), "Give me, I pray thee, of thy son's mandrakes." In later times the bryony became substituted for the mandrake; and it has since continued to form a spurious, profitable article with mountebank doctors. In Henry the Eighth's day, ridiculous little images made from the bryony roots, and called puppettes, or mammettes, were accredited with magical powers, and fetched high prices with the simple. Italian ladies have been known to pay as much as thirty golden ducats for one of these artificial mandrakes. Unscrupulous vendors of the fraudulent articles used to find a young, thriving bryony plant, and to open the earth round it. Then, being prepared with a mould, such as is used for making plaster of Paris figures, they fixed it close to the root, and fastened it with wires to keep it in place. Afterwards, by filling up the earth to the root, they left it to assume the required shape, which was generally accomplished in a single summer.

The black bryony (Tamus vulgaris), which also grows abundantly in our English hedges, is quite a distinct plant, and belongs to the natural order of yams. It possesses cordate smooth leaves, and produces scarlet berries, which are elliptical in shape, and larger than those of the white bryony. A tincture is made with spirit of wine from the fresh rootstock, which is of great value for unbroken chilblains when applied externally as a lotion. This plant is called black bryony from its dark leaves and black root.

Reverting to the white bryony, I would say that its medicinal tincture is of special service to persons of dark hair and complexion, with firm, fleshy fibre, and of bilious, irritable temperament. Also, it is particularly valuable for relieving coughs and colds caused by exposure to east winds. Quite on the contrary, the catarrhal troubles of young children and of sensitive females are better met by ipecacuanha. But this medicament, if pressed at all too far, will cause nausea.

> "Sitting in a shady grove
> With my Juliana,
> Lozenges I gave my love—
> Ipecacuanha.
>
> Full twenty from the lozenge box
> The foolish nymph did pick;
> Then, sighing gently, said to me,
> "My Damon, I am sick."

